# piRNA-31115 Promotes Cell Proliferation and Invasion via PI3K/AKT Pathway in Clear Cell Renal Carcinoma

**DOI:** 10.1155/2021/6915329

**Published:** 2021-11-08

**Authors:** Xinghua Du, Haomin Li, Xuexia Xie, Liping Shi, Fan Wu, Guoliang Li, Caiyong Lai, Baoli Heng

**Affiliations:** ^1^Department of Urology, The First Affiliated Hospital of Jinan University, Guangzhou, China; ^2^Department of Urology, The Sixth Affiliated Hospital of Jinan University, Dongguan, China; ^3^Yingde Center, Institute of Kidney Surgery, Jinan University, Guangdong, China; ^4^Postdoctoral Mobile Station, The First Clinical Medical College of Jinan University, Guangzhou, China; ^5^Department of Urology, People's Hospital of Yingde City, Yingde, China

## Abstract

PIWI-interacting RNAs (piRNAs) are small noncoding RNAs that play important roles in germline development and carcinogenesis. In this study, we used the deep sequencing of small RNA Transcriptome to explore the piRNA expression in six clear cell renal carcinoma (ccRCC) tissues and matched adjacent normal tissues and found that six piRNAs were upregulated and sixteen were downregulated in ccRCC tissues. Among them, piRNA-31115 (NCBI accession number: DQ571003) was the most upregulated piRNA in ccRCC tissues compared with matched adjacent normal tissues. Quantitative real-time PCR (qRT-PCR) was used to confirm piR-31115 expression in other ccRCC tissues (*n* = 40) and ccRCC cell lines. Besides, function analysis demonstrated that silencing of piR-31115 inhibited ccRCC cell proliferation, motility, and invasiveness. Mechanistic investigations showed that piRNA-31115 may activate epithelial-mesenchymal transition (EMT) via the PI3K/AKT signaling pathway. Hence, piR-31115 may represent an oncogene in the development of ccRCC.

## 1. Introduction

Renal cell carcinoma (RCC) is one of the most common malignant tumors of the urinary system, representing approximately 2% to 3% of all adult malignancies [1, 2]. Clear cell renal cell carcinoma (ccRCC) is the most common type of RCC, which accounts for 60% to 85% of all RCC cases [3]. Current imaging methods, such as computed tomography (CT), have been able to detect more and more small renal masses; however, ccRCC is insidious, and about 30% of patients will develop local invasion and distant metastatic lesions even after they are found [4, 5]. Hence, these ccRCC patients have already lost their chances of radical surgical resection and exhibit a low five-year survival rate. Therefore, a better prediction of ccRCC patients to potential personalized therapeutic strategies is urgently needed.

piRNAs are a class of small noncoding RNAs of 24-30 nucleotides that can bind to PIWI proteins from the Argonaute family to accomplish their regulatory function, resulting in the formation of a silencing protein complex that silences complementary sequences [6]. On the one hand, the PIWI-piRNA complex can silence the transposable elements (TEs) to maintain the germline genome integrity at the transcriptional and posttranscriptional level, which is correlated with its biogenesis and may be a conserved function among different species [7]. On the other hand, the PIWI-piRNA complex has been primarily described as functioning to the development of cancer through epigenetic modifications like DNA methylation and histone modifications [8]. Yan et al. revealed that piR-823 was a direct regulator of DNA methyltransferases 3A and 3B (DNMT3A and DNMT3B); the inhibition of piR-823 decreased global methylation in multiple myeloma and restored the methylation of a CpG island in the promoter of p16 [9]. Taken together, piRNAs play an important role in the regulation of gene expression via TE silencing and epigenetic modifications.

According to numerous studies, piRNAs have abundant biological functions and are involved in various physiological and pathological processes of cancers, such as multiple myeloma [9], bladder cancer [10], breast cancer [11], and gastric cancer [12]. These findings indicate that piRNAs may serve as novel prognostic biomarkers and may represent a significant therapeutic target in tumors [13]. In this study, we used small RNA deep sequencing to explore the effects of piRNA in ccRCC and identified a novel piRNA, piR-31115, which has not been reported as an oncogene in the development of ccRCC.

## 2. Methods

### 2.1. Human ccRCC Tissue Samples

All of the ccRCC tissues and matched adjacent normal renal tissues were obtained from patients who underwent tumorectomies at the Department of Urology of the First Affiliated Hospital of Jinan University (Guangzhou Overseas Chinese Hospital; Guangzhou, China) between 2014 and 2018. After the surgery, we collected normal kidney tissues ≥ 2 cm away from the tumor. All of the samples were placed in RNA-later and refrigerated at 4°C overnight, after which they were frozen in liquid nitrogen. At least two clinical pathologists confirmed the histological and pathological diagnoses. All of the samples were obtained with appropriate informed consent from the patients and were approved by the Ethical Committee of the First Affiliated Hospital of Jinan University.

### 2.2. Small RNA Library Preparation, Sequencing, and Data Analysis

A total amount of 3 *μ*g total RNA per sample was used as input material for the small RNA library. Sequencing libraries were generated using the NEBNext®Multiplex Small RNA Library Prep Set for Illumina® (NEB, USA) following the manufacturer's recommendations, and index codes were added to attribute sequences to each sample. Briefly, an NEB 3′ SR adaptor was directly and specifically ligated to the 3′ end of miRNAs, siRNAs, and piRNAs. After the 3′ ligation reaction, the SR RT primer hybridized to the excess of the 3′ SR adaptor (that remained free after the 3′ ligation reaction) and transformed the single-stranded DNA adaptor into a double-stranded DNA molecule. This step is important to prevent adaptor-dimer formation. Besides, dsDNAs are not substrates for ligation mediated by T4 RNA Ligase 1 and, therefore, do not ligate to the 5′ SR adaptor in the subsequent ligation step. A 5′-end adapter was ligated to the 5′ ends of miRNAs, siRNAs, and piRNAs. Then, the first strand cDNA was synthesized using M-MuLV Reverse Transcriptase (RNase H–). PCR amplification was performed using the LongAmp Taq 2x Master Mix, SR Primer for Illumina, and Index (X) Primer. PCR products were purified on 8% polyacrylamide gel (100 V, 80 min). DNA fragments corresponding to 140~160 bp (the length of small noncoding RNA plus the 3′ and 5′ adaptors) were recovered and dissolved in the 8 *μ*l elution buffer. Finally, library quality was assessed on the Agilent Bioanalyzer 2100 system using DNA High-Sensitivity Chips. The clustering of the index-coded samples was performed on a cBot Cluster Generation System using the TruSeq SR Cluster Kit v3-cBot-HS (Illumina) according to the manufacturer's instructions. After cluster generation, the library preparations were sequenced on an Illumina Hiseq 2500/2000 platform, and 50 bp single-end reads were generated.

Raw data (raw reads) of fastq format were firstly processed through custom perl and python scripts. Adaptor sequences and low-quality reads were removed to obtain clean reads from raw reads. Small RNA tags were mapped to piRNABank (http://pirnabank.ibab.ac.in/) to screen and annotate piRNAs using Bowtie1. Normalization and test for differential piRNA expression between tumor and matched normal renal tissues were performed using the DESeq2 R/Bioconductor package.

### 2.3. Culturing and Treatment of Cell Lines

Human ccRCC cell lines (CAKi-1, CAKi-2, 786O, ACHN, and 769P) and the immortalized normal renal epithelial cell line, HK-2, were purchased from the American Type Culture Collection (ATCC, Manassas, USA). 786O, ACHN, and 769P were maintained in RPIM-1640 medium (Biological Industries, Israel) supplemented with 10% fetal bovine serum (FBS; Biological Industries, Israel) and 1% penicillin/streptomycin (Biological Industries, Israel). CAKi-1 and CaKi-2 cells were cultured in 5A medium (Biological Industries, Israel) supplemented with 10% FBS and 1% penicillin/streptomycin. HK-2 cells were cultured in DMEM/F-12 medium (Biological Industries, Israel) supplemented with 10% FBS and 1% penicillin/streptomycin. All of the cells were cultured in a humidified incubator (Thermo Fisher Scientific, USA) with 5% CO_2_ at 37°C.

### 2.4. RNA Extraction, Reverse Transcription PCR (RT-PCR), and Quantitative Real-Time PCR (qRT-PCR)

Total RNA was separated by TRIzol (Life Technologies, USA) according to the manufacturer's instructions. For RT-PCR, RNA was reverse transcribed to cDNA with random primers using the miScript II RT Kit (Qiagen, Hilden, Germany). The RT-PCR reactions were performed using a Bio-Rad T100 PCR (Bio-Rad, USA). To quantify the amount of piRNAs, qRT-PCR was performed using the Qiagen miScript SYBR Green PCR technology (Hilden, Germany). U6 was used as an internal control. All of the analyses were performed using the ABI 7500 quantitative real-time PCR instrument (Life Technologies, USA). The 2^−ΔΔCT^  method was used to calculate the relative expression levels of different genes. The primer sequence of piR-31115 is 5′-AGCCTGAGCAACATAGCGAG-3′.

### 2.5. Cell Transfection

The piR-31115 inhibitor and the control inhibitor were synthesized by Gema (Shanghai, China). The knockdown of piR-31115 expression was achieved through piR-31115 inhibitor (5 nmol) transfection using Lipofectamine 2000 (Invitrogen, USA) according to the manufacturer's instructions, and a control inhibitor was used as a control. Cells were collected 48 h after transfection. PiR-31115 expression levels were determined by qRT-PCR.

### 2.6. CCK-8 Assay

The cell proliferation ability was evaluated by the Cell Counting Kit-8 (CCK-8; Dojindo, Japan). Approximately 3 × 10^3^ transfected CaKi-1 and 786O cells were cultured in 96-well plates. At 0, 24, 48, and 72 h, 10 ml of CCK-8 reagent was added to each well and then incubated at 37°C for 2 h. The optical density at 450 nm of every well was tested by an automatic microplate reader (Thermo Fisher Scientific, USA). All assays were repeated at least three times.

### 2.7. Wound Healing Assay

CaKi-1 and 786O cells were cultured in 6-well plates and scratched by 20 *μ*l pipette tips after reaching 80–90% confluency. The cells were cultured in an incubator at 37°C for 24 h with serum-free 5A or RPIM-1640 medium and were imaged at 0 h and 24 h via an Olympus IX51 microscope (Olympus, Japan).

### 2.8. Transwell-Migration and Invasion Assays

Approximately 5 × 10^4^ transfected CaKi-1 and 786O cells were placed into the upper chambers of 24-well plates (Corning, NY, USA) with or without Matrigel (BD Biosciences, NY, USA), while 500 *μ*l of medium with 20% FBS was placed in the lower chambers to act as a chemoattractant. The chambers were incubated at 37°C for 24 h, cells were fixed with 4% paraformaldehyde, and were then stained with 0.1% crystal violet for 30 min. The number of cells that migrated or invaded was calculated in five random fields under an Olympus IX51 inverted microscope (Olympus, Japan). All assays were repeated at least three times.

### 2.9. Western Blot

Total protein was extracted from cells using RIPA lysis buffer (Keygen Biotech). The lysate protein was separated by 10% SDS-PAGE and electrophoretically transferred onto polyvinylidene difluoride (PVDF) membranes (Millipore, Bedford, MA, USA). After blocking with 5% nonfat milk in TBST for 2 h, proteins were then incubated with primary and secondary antibodies. Signal detection was visualized by using enhanced chemiluminescence. The concentration was determined using a bicinchoninic acid (BCA) protein assay kit (Pierce, Thermo Fisher Scientific). The primary antibodies used for the western blots were as follows: rabbit antibodies against vimentin (Cell Signaling Technology), GAPDH (Cell Signaling Technology, 5174), snail (Cell Signaling Technology, 3879), E-cadherin (BD Biosciences, San Jose, CA, USA, 564186), phospho-Akt (Cell Signaling Technology, 4060), Akt (Cell Signaling Technology, 4691), PI3K (Cell Signaling Technology, 4257), and phospho-PI3K (Cell Signaling Technology, 17366). Related data were analysed with Image Lab software.

### 2.10. Statistical Analysis

All of the data are shown as the mean ± SD. Statistical analyses were performed using SPSS 22.0 statistical software (SPSS, Chicago, USA) and GraphPad Prism 7.0. Differences between ccRCC tissues and paired adjacent normal tissues and between paired preoperative and postoperative OS patient samples were analyzed using the Student's *t*-test. *P* values < 0.05 were considered statistically significant.

## 3. Results

### 3.1. Small RNA Sequence Revealed piR-31115 Was Upregulated in ccRCC

**Figure 1:**
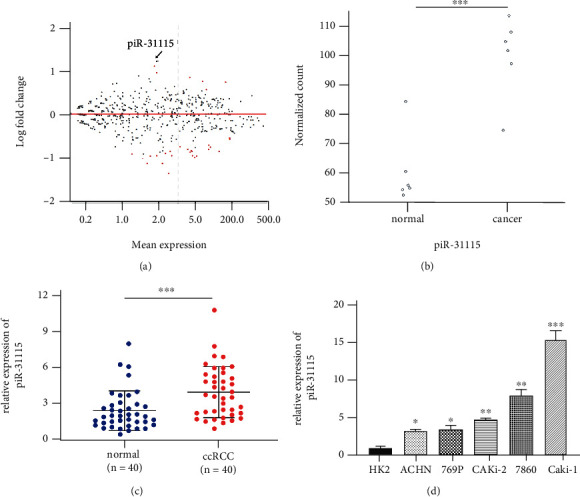
piRNA expression profiling results and confirmation of piR-31115 upexpression in ccRCC relative to normal kidney tissue. (a) Results of the deep sequencing of small RNA transcriptome profiling in ccRCC relative to normal renal tissue specimens. piRNAs with detectable expression levels are plotted according to average log2 in each type. piR-31115 (NCBI accession number: DQ571003) was labeled. (b) The normalized count of piR-31115 in the deep sequencing of the small RNA transcriptome. (c) Validation of piR-31115 expression levels in another 40 ccRCC tissues vs. adjacent normal renal tissue specimens by qPCR. (d) Measurement of piR-31115 expression in immortalized normal renal epithelial cell line (HK-2) and human ccRCC cell lines (ACHN, 769P, CaKi-2, 786O, and CaKi-1) by qPCR. ^∗^*P* < 0.05; ^∗∗^*P* < 0.01; ^∗∗∗^*P* < 0.001; the data are presented as the means ± SD based on triplicate independent experiments.

The deep sequencing of small RNA transcriptomes was used to explore the piRNA expression in six pairs of clear cell renal carcinoma tissues and matched adjacent normal tissues. In total, 19 piRNAs were upregulated and 41 piRNAs were downregulated in ccRCC tissues (fold-changes ≥ 2 and *P* value < 0.05); all the piRNAs are listed in the supplementary materials. Among them, piRNA-31115 was the most upregulated piRNA in ccRCC tissues compared with matched adjacent normal tissues (Figure 1(a)). Thus, we selected piR-31115 for further investigation.

We then validated the expression level of piR-31115 in another 40 pairs of ccRCC tissues and matched adjacent normal renal tissues via qRT-PCR. As shown in Figure 1(c), piR-31115 was upregulated in most ccRCC tissues, which was consistent with the small RNA sequence data (Figure 1(b)). Next, we examined the expression of piR-31115 in five ccRCC cell lines (786O, 769P, ACHN, CAKi-1, and CAKi-2) and the immortalized normal renal epithelial cell line (HK-2). As shown in Figure 1(d), piR-31115 was upregulated in five ccRCC cell lines compared with HK-2 (Figure 1(d)).

### 3.2. Silence of piR-31115 Inhibits Cell Proliferation of ccRCC Cells *In Vitro*

**Figure 2:**
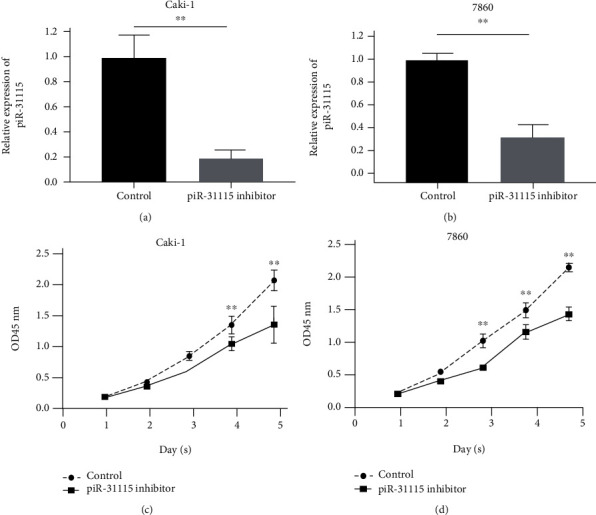
Reduction of ccRCC cell proliferation by piR-31115. (a, b) The expression levels of piR-31115 in CaKi-1 and 786O cells after transfection of the piR-31115 inhibitor and the control inhibitor were tested by qRT-PCR in vector and circHIPK3-overexpression groups. (c, d) Cell proliferation of CaKi-1 and 786O cells transfected with the piR-31115 inhibitor and the control inhibitor was examined by a Cell Counting Kit-8 (CCK-8) assay at different timepoints. ^∗^*P* < 0.05; ^∗∗^*P* < 0.01; ^∗∗∗^*P* < 0.001; the data are presented as the means ± SD based on triplicate independent experiments.

To explore the effect of piR-31115 in ccRCC carcinogenesis, CaKi-1 and 786O cells were transfected with the piR-31115 inhibitor or the control inhibitor RNA. qRT-PCR results suggested that transfection of the piR-31115 inhibitor could decrease the expression of piR-31115 compared with the control group (Figures 2(a) and 2(b)).

Next, we further examined the proliferation of ccRCC cell lines. As shown in Figures 2(b)–2(d), a CCK-8 assay indicated that the silence of piR-31115 inhibits significantly inhibited ccRCC cellular growth compared with that of the control group. This result revealed that the silence of piR-31115 inhibited proliferation of CaKi-1 and 786O cells *in vitro*.

### 3.3. Silence of piR-31115 Inhibits Invasion and Metastasis of ccRCC Cell Lines *In Vitro*

**Figure 3:**
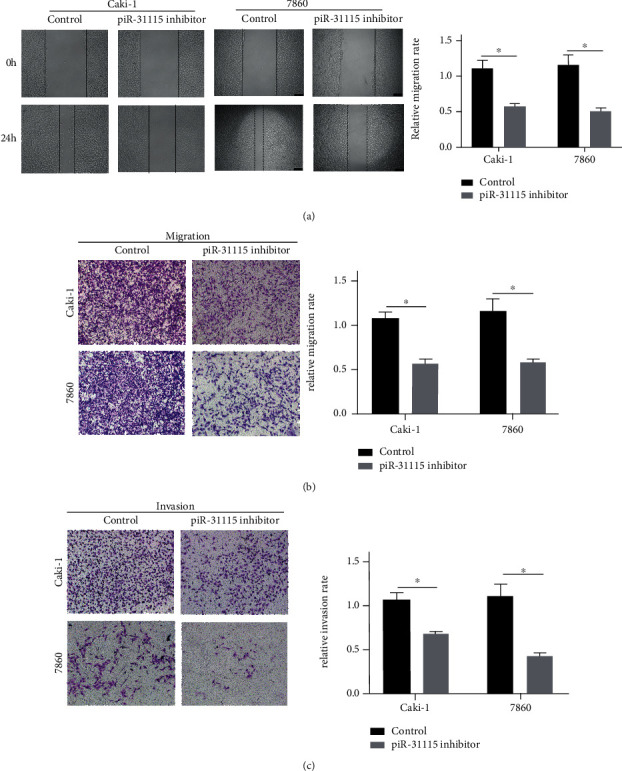
Knockdown of piR-31115 inhibits invasion and metastasis of ccRCC cell lines *in vitro*. (a) The effect of piR-31115 on migratory capability was evaluated by a wound-healing assay in CaKi-1 and 786O cells. (b, c) The effects of piR-31115 in migratory and invasive capabilities were evaluated by transwell-migration and Matrigel-invasion assays in 786O and CaKi-1 cells. ^∗^*P* < 0.05; ^∗∗^*P* < 0.01; ^∗∗∗^*P* < 0.001; the data are presented as the means ± SD based on triplicate independent experiments.

Next, we assayed the invasion and metastasis of ccRCC cells. A wound-healing assay suggested that silence of piR-31115 suppressed cellular migration in CaKi-1 and 786O cells (Figure 3(a)). Similarly, silence of piR-31115 also inhibited migration and invasion of ccRCC cells in transwell-migration and Matrigel-invasion assays (Figures 3(b) and 3(c)). These findings indicate that the silence of piR-31115 inhibited migration and invasion of ccRCC cells *in vitro*.

### 3.4. piR-31115 May Activate Epithelial-Mesenchymal Transition Process and the PI3K/AKT Signaling Pathway

**Figure 4:**
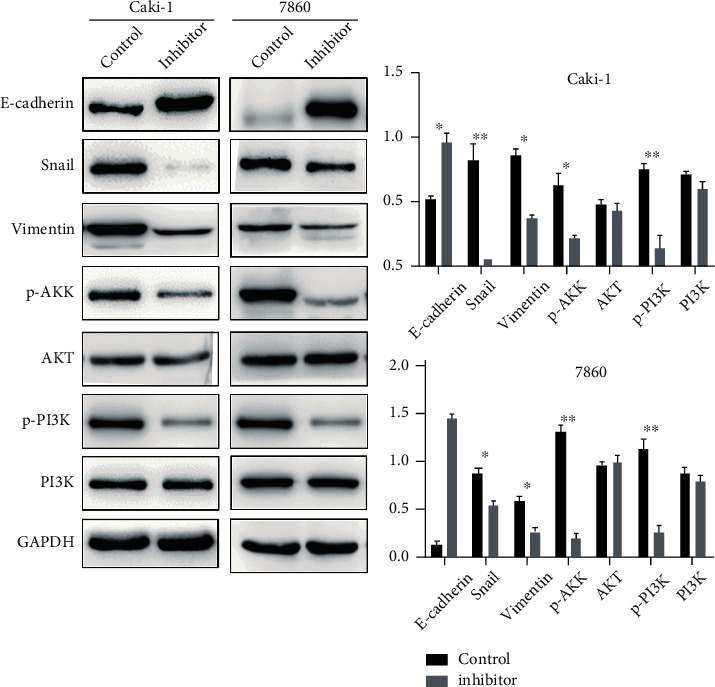
piR-31115 may activate the epithelial-mesenchymal transition process and the PI3K/AKT signaling pathway. (a) The protein expression of E-cadherin, snail, vimentin, pAKT, AKT, p-PI3K, and PI3K was detected by western blotting after knockdown of piR-31115 in CaKi-1 and 786O cells. Protein expression calculated by ImageJ is shown on the right. ^∗^*P* < 0.05; ^∗∗^*P* < 0.01; ^∗∗∗^*P* < 0.001; the data are presented as the means ± SD based on triplicate independent experiments.

Epithelial-mesenchymal transition (EMT) is a process in which epithelial cells acquire mesenchymal features. In cancer, EMT is associated with tumor initiation, invasion, metastasis, and resistance to therapy. Notably, after downregulating piRNA-31115 levels, the protein expression of epithelial marker E-cadherin was upregulated and mesenchymal markers vimentin and snail decreased (Figure 4(a)). Emerging evidence has shown that PI3K is a key oncogenic factor involved in the activation of several pathways, including cell proliferation and invasion, as well as the EMT process. Western blot was performed to determine pPI3K/PI3K and pAKT/AKT level in CaKi-1 and 786O cells in response to piR-31115 inhibitor treatment (Figure 4(a)). We found that phosphorylation of AKT and PI3K expression was significantly reduced after knockdown of piR-31115. The results suggest that piR-31115 may activate the epithelial-mesenchymal transition process and the PI3K/AKT signaling pathway in ccRCC.

## 4. Discussion

piRNA was first discovered and identified as a new long small interfering RNA (siRNA) in *Drosophila melanogaster* and later found highly abundant in mouse testis and associated with PIWI protein from the Argonaute family [14, 15]. Currently, it is conservatively estimated that there are approximately 20,000 piRNAs on the genome of eukaryotes. Mature piRNAs could bind with PIWI proteins and form a complex in the germline, which may reach their target transcripts and mobilize the silencing machinery to the block transcription of transposable elements (TE), maintaining genome integrity. For example, it was found that the piRNA-PIWI complex locates the transcription site by recognizing the primary transcript of the transposon in the follicular cells of *Drosophila*, and then recruits histone methyltransferase to perform H3K9 methylation modification of H1 histone to inhibit transcription [8].

Unlike miRNA, piRNA has not been widely studied in cancer; only a few studies have shown that the expression profile of piRNA has changed in cancer. However, with the small RNA deep sequencing used for comparing different expression profiles in different tissues, it has been suggested that piRNA-PIWI have kinds of biological functions in various malignant solid tumors [6, 16, 17]. Cheng et.al. reported that the expression of piR-823 is significantly reduced in the peripheral blood of patients with gastric cancer and gastric cancer tissues, indicating that piR-823 has potential as a diagnostic marker for gastric cancer, and the expression level is related to the stage of gastric cancer [12]. Overexpression of piR-823 will inhibit gastric cancer cell growth. In addition, piR-823 has also been shown to be associated with ccRCC [18].

Previous studies had revealed that piR-32051, piR-39849, and piR-43607 were upregulated in ccRCC [19, 20], whereas other piRNAs like piR-823, piR-38756, piR-57125, piR-34536, and piR51810 were downregulated in tumor tissues [16, 21]. In this study, we performed small RNA deep sequencing to investigate the comprehensive piRNA expression in ccRCC and found that piR-31115 was the most upregulated piRNA in ccRCC tissues compared with matched adjacent normal tissues. Moreover, silencing of piR-31115 inhibited ccRCC cell proliferation, mobility, and invasiveness.

In addition, a large number of studies have found that human PIWI protein has abnormal expression in a variety of tumors, such as breast cancer [11], pancreatic cancer [22], and liver cancer [23], but how the abnormal expression of PIWI protein affects the clinical characteristics of tumor is unknown. A previous study reported that the upregulation of piR-Hep in hepatocellular carcinoma could promote hepatocellular cell proliferation via binding with PIWI2/HILI to affect PI3K/AKT signaling [17]. Here, we also found that piR-31115 may activate the epithelial-mesenchymal transition process and the PI3K/AKT signaling pathway in ccRCC, but which PIWI protein interacts with piR-31115 needs further study. Some studies of piRNA function have indicated that piRNAs could target mRNA transcripts to degrade or inhibit tumor suppressor genes or oncogenes, respectively, which was a distinctive mechanism of miRNA. However, it needs further investigation.

## 5. Conclusions

In summary, we explored the piRNA expressions in six pairs of ccRCC tissues and found that piR-31115 was upregulated and promoted cell proliferation and invasion via the EMT process and the PI3K/AKT signaling pathway in ccRCC. piR-31115 may be a potential therapeutic target in ccRCC patients. However, there are still some inherent limitations in this study: We did not investigate the effect of biological function of piR-31115 *in vivo*. The mechanism of piR-31115 is still largely unknown. Given that, further investigations are still required in animal models and in the molecular mechanisms of the other signaling pathways.

## Data Availability

All data included in this study are available upon request by contact with the corresponding author.

## Supplementary Materials

Supplementary Materials Table S1: differentially expressed piRNAs detected by small RNA sequencing in ccRCC tissues and matched adjacent normal renal tissues (fold − changes ≥ 2 and *P* value < 0.05) (baseMean: mean of normalized counts for all samples; log2FoldChange: log2 fold change; lfcSE: standard error; *P* value: Wald's test *P* value; *p*adj: BH adjusted *P* values).
