# Supplementary material for: piRNA-31115 Promotes Cell Proliferation and Invasion via PI3K/AKT Pathway in Clear Cell Renal Carcinoma
Source: Dis Markers. 2021 Nov 8;2021:6915329. doi: 10.1155/2021/6915329 (PMC8592738; doi:10.1155/2021/6915329)
Supplement: Supplementary Materials — Table S1: differentially expressed piRNAs detected by small RNA sequencing in ccRCC tissues and matched adjacent normal renal tissues (fold − changes ≥ 2 and P value < 0.05) (baseMean: mean of normalized counts for all samples; log2FoldChange: log2 fold change; lfcSE: standard error; P value: Wald's test P value; padj: BH adjusted P values). [file 6915329.f1.docx]

**Supplementary Table**

**Table S1. Differentially expressed piRNAs detected by Small RNA sequencing in ccRCC tissues and matched adjacent normal renal tissues (fold-changes ≥ 2 and P value < 0.05).** (baseMean: mean of normalized counts for all samples; log2FoldChange: log2 fold change; lfcSE: standard error; pvalue: Wald test p-value; padj: BH adjusted p-values)

| NCBI number | baseMean | log2FoldChange | lfcSE | PValue | padj |
| --- | --- | --- | --- | --- | --- |
| DQ570812.1 | 3.657352225 | 1.147387945 | 0.381141734 | 0.002609064 | 0.049709533 |
| DQ596538.1 | 4.046931278 | 0.98490383 | 0.371968077 | 0.008101309 | 0.091646055 |
| DQ571333.1 | 1.861327677 | 0.920820725 | 0.383085929 | 0.016230425 | 0.158794969 |
| DQ598378.1 | 16.61027937 | 0.874142503 | 0.295032219 | 0.003047839 | 0.052606408 |
| DQ596531.1 | 2.899555507 | 0.848248156 | 0.383617914 | 0.027023393 | 0.222328826 |
| DQ571419.1 | 28.51914947 | 0.791655178 | 0.241738385 | 0.001057208 | 0.030657087 |
| DQ596744.1 | 3.042928811 | 0.779355084 | 0.381851998 | 0.041252088 | 0.281759545 |
| DQ571003.1 | 75.78437866 | 1.762952726 | 0.174288993 | 1.20E-05 | 0.004345446 |
| DQ573682.1 | 0.728644855 | 0.738591483 | 0.324264286 | 0.022741751 | NA |
| DQ590642.1 | 0.615698597 | 0.728694011 | 0.325284113 | 0.02507943 | NA |
| DQ573352.1 | 8.608836694 | 0.666979734 | 0.311246764 | 0.032118795 | 0.238690426 |
| DQ570326.1 | 33.43772454 | 0.586804211 | 0.210526695 | 0.005314679 | 0.073996689 |
| DQ584904.1 | 14.31271859 | 0.579270877 | 0.293877101 | 0.048708655 | 0.309989263 |
| DQ582302.1 | 27.62517429 | 0.558833123 | 0.219144997 | 0.010770406 | 0.114673141 |
| DQ584545.1 | 12.5288891 | 0.551564327 | 0.274791206 | 0.044727048 | 0.294385296 |
| DQ570698.1 | 31.99613863 | 0.47630371 | 0.209969144 | 0.023302024 | 0.20084125 |
| DQ592970.1 | 88.74703037 | 0.375199052 | 0.165364972 | 0.0232735 | 0.20084125 |
| DQ593048.1 | 62.05781715 | 0.372452229 | 0.173996949 | 0.032308925 | 0.238690426 |
| DQ571500.1 | 45.14672677 | 0.359393174 | 0.182410532 | 0.048810464 | 0.309989263 |
| DQ597974.1 | 59.70228127 | 0.345675046 | 0.16575838 | 0.037031628 | 0.262851947 |
| DQ571243.1 | 60.31395364 | 0.362709227 | 0.165814908 | 0.028710831 | 0.230962684 |
| DQ596467.1 | 102.6421948 | 0.412301688 | 0.199301623 | 0.038571222 | 0.268515049 |
| DQ596466.1 | 98.78291277 | 0.421563943 | 0.198240547 | 0.033459375 | 0.242245879 |
| DQ597401.1 | 27.05673144 | -0.425485906 | 0.211535456 | 0.044281483 | 0.294385296 |
| DQ596469.1 | 103.8706218 | -0.431183764 | 0.198784172 | 0.030074704 | 0.236674848 |
| DQ596470.1 | 110.3570736 | -0.454011091 | 0.197016317 | 0.021198296 | 0.191844575 |
| DQ570001.1 | 21.10482995 | -0.563813191 | 0.250045861 | 0.024143428 | 0.203253976 |
| DQ597975.1 | 91.08132014 | -0.574675156 | 0.185679068 | 0.001968185 | 0.041910757 |
| DQ576872.1 | 91.19870496 | -0.595375826 | 0.186777586 | 0.001434493 | 0.034619098 |
| DQ598675.1 | 37.35795586 | -0.746340678 | 0.251931357 | 0.003051753 | 0.052606408 |
| DQ600078.1 | 0.68858483 | -0.779451035 | 0.325025755 | 0.016479428 | NA |
| DQ597163.1 | 10.92600238 | -0.781202304 | 0.294955453 | 0.008083948 | 0.091646055 |
| DQ596309.1 | 5.356061966 | -0.790029127 | 0.367651359 | 0.031645968 | 0.238690426 |
| DQ597886.1 | 39.70589478 | -0.79636668 | 0.285469186 | 0.005276073 | 0.073996689 |
| DQ593325.1 | 20.86560819 | -0.801646706 | 0.339460887 | 0.018199752 | 0.168931028 |
| DQ593275.1 | 37.01735407 | -0.840450681 | 0.29207747 | 0.004008496 | 0.0647302 |
| DQ591357.1 | 15.20197094 | -0.842506428 | 0.278215111 | 0.002459701 | 0.049467313 |
| DQ584698.1 | 17.62873092 | -0.878162277 | 0.270004055 | 0.001144332 | 0.030657087 |
| DQ584699.1 | 5.20058142 | -0.884928095 | 0.371152188 | 0.017112908 | 0.163022963 |
| DQ579193.1 | 57.65697027 | -0.895483852 | 0.248091641 | 0.000306802 | 0.022212444 |
| DQ584697.1 | 7.156924313 | -0.897718548 | 0.331416242 | 0.00675404 | 0.090537359 |
| DQ570849.1 | 18.2575448 | -0.897980019 | 0.246556014 | 0.00027043 | 0.022212444 |
| DQ596738.1 | 4.641972312 | -0.910599657 | 0.361490811 | 0.011768468 | 0.121719582 |
| DQ582262.1 | 16.74650397 | -0.913771593 | 0.262971387 | 0.000511244 | 0.023537159 |
| DQ596225.1 | 5.87475682 | -0.946185869 | 0.353323774 | 0.007407273 | 0.090537359 |
| DQ582201.1 | 1.40892078 | -0.95776398 | 0.356311649 | 0.007188244 | 0.090537359 |
| DQ596310.1 | 2.301930602 | -0.958264163 | 0.384919505 | 0.012791632 | 0.128626968 |
| DQ600952.1 | 7.673143136 | -0.990550377 | 0.345411309 | 0.00413413 | 0.0647302 |
| DQ593292.1 | 4.050589124 | -0.994019447 | 0.378353999 | 0.008608549 | 0.094433171 |
| DQ597110.1 | 18.55122165 | -0.996615882 | 0.267338043 | 0.000193064 | 0.022212444 |
| DQ591244.1 | 22.30776382 | -1.001527512 | 0.308892821 | 0.001185633 | 0.030657087 |
| DQ596275.1 | 3.058017666 | -1.009861877 | 0.377709234 | 0.007503096 | 0.090537359 |
| DQ591243.1 | 20.47816113 | -1.043255855 | 0.305961557 | 0.000650198 | 0.023537159 |
| DQ582264.1 | 11.76185875 | -1.060974281 | 0.313614255 | 0.000716843 | 0.023590641 |
| DQ582265.1 | 11.92174613 | -1.068434455 | 0.304691372 | 0.00045385 | 0.023537159 |
| DQ598107.1 | 2.221205737 | -1.074871135 | 0.376368639 | 0.004291505 | 0.0647302 |
| DQ596354.1 | 2.682982412 | -1.205497238 | 0.38478324 | 0.001730733 | 0.039157845 |
| DQ596311.1 | 5.168417055 | -1.211320977 | 0.354353193 | 0.000629904 | 0.023537159 |
| DQ600079.1 | 5.168417055 | -1.211320977 | 0.354353193 | 0.000629904 | 0.023537159 |
| DQ598274.1 | 6.719590122 | -1.440965591 | 0.37915732 | 0.000144438 | 0.022212444 |
